# Oncology in Iraq’s Kurdish Region: Navigating Cancer, War, and Displacement

**DOI:** 10.1200/JGO.2016.008193

**Published:** 2017-04-27

**Authors:** Mac Skelton, Layth Y.I. Mula-Hussain, Kadhim Faruq Namiq

**Affiliations:** **Mac Skelton**, The Institute of Regional and International Studies, American University of Iraq, Sulaimani; **Layth Y.I. Mula-Hussain**, Zhianawa Cancer Center; and **Kadhim Faruq Namiq**, Hiwa Cancer Hospital, Sulaymaniyah, Kurdistan, Iraq; and **Mac Skelton**, Johns Hopkins University, Baltimore, MD.

## Introduction

On October 20, 2016, oncologists gathered in Erbil for the first Best of ASCO Meeting (officially licensed by the American Society of Clinical Oncology [ASCO]) to take place in the Kurdish region of Iraq, which is governed by the semiautonomous Kurdistan Regional Government (KRG).^1^ The meeting was one more indication of the gradual progress of the KRG oncology system, which is only 10 years in the making. The KRG and local charities have invested heavily in the public health care and oncology sector during the past decade. Hiwa Cancer Hospital (HCH), located in the Kurdish city of Sulaymaniyah, was opened in 2007 and eventually became the second largest public provider of cancer care in all of Iraq (after Al-Amal National Cancer Center in Baghdad). This commentary will discuss the history and development of public oncology in the Kurdish region of Iraq during the past decade and address emerging trends. In particular, we discuss the influx of war-displaced patients with cancer into the Kurdish region since 2014 (largely from areas under the Islamic State of Iraq and Syria [ISIS] control and/or opposition militia groups). We lay out an interdisciplinary research agenda to understand the complex dynamics of cancer care for this patient population.

## The KRG As an Emerging Cancer Hub

Until recently, it would have been unthinkable to place Baghdad and Sulaymaniyah in the same category in terms of cancer services. Baghdad was and remains the seat of the national health care system of Iraq, which once was considered among the strongest in the Middle East region.^2^ Historically, the high-level health care and oncology centers in Iraq were based in centrally located Baghdad and in the northern city of Mosul; thus, patients in the Kurdish region typically traveled domestically for chemotherapy and radiotherapy. The reliance on interprovincial referrals to Baghdad and Mosul became increasingly untenable because of the effects of 13 years of United Nations sanctions (from 1990 to 2003).^3^ Sanctions placed severe restrictions on imports and rendered hospital maintenance impossible. Even the nation’s top cancer care centers struggled to keep medications and equipment adequately stocked to meet the high volume of patients from across the country.^4^ Additional problems developed after the US-led invasion in 2003. Urban warfare blurred the lines between civilian and combatant spaces,^5^ which compelled a mass exodus of doctors to neighboring countries.^6^

As conditions of insecurity compromised health care services throughout much of the country between 2004 and 2007,^7^ a favorable power-sharing agreement allowed the KRG to enjoy a period of relative political stability and economic growth. Ultimately, these conditions enabled the establishment and development of new KRG cancer hospitals. Many doctors from throughout Iraq relocated to the KRG and strengthened the local ranks of specialists. New cancer-related programs, initiatives, and centers were announced.

Approximately 3,000 newly diagnosed patients with hematologic and oncologic diseases are admitted or referred to different departments of HCH each year. Palliative care and bone marrow transplant units were introduced recently at HCH. In late 2016, an Italian team visited the city and signed a contract with the Sulaymaniyah Directorate of Health and HCH to initiate the bone marrow transplantation center in Sulaymaniyah. The public radiotherapy center in the city, Zhianawa Cancer Center (ZCC), is the only Union for International Cancer Control member in Iraq and has implemented the first accredited board-training program for radiation oncology in the country, which includes brachytherapy and intensity-modulated radiation therapy services.

The conditions of cancer research have improved greatly in the KRG during the last 4 years. Until recently, there was no organized cancer registration program in the KRG.^8^ In September 2013, however, the Federal Ministry of Health and Iraqi Cancer Registry (founded in 1976) formally opened sectors in the three governorates of the KRG (Sulaymaniyah, Erbil, and Dohuk). These sectors send cancer-related data directly to the central registry in Baghdad three times annually and provide the same data to KRG health authorities. The incorporation of the KRG into the national registry has encouraged enhancement of hospital data systems. The recently installed data portal at HCH has attracted both domestic and international researchers to perform (mostly retrospective) studies.

## Displacement and Cancer Care

These advances in cancer care and research should be applauded; however, we contend that one of the most important next steps will be the development of enhanced frameworks for the administration of care for war-displaced patients. A total of 1.8 million Syrian and internally displaced Iraqis now reside in the KRG.^9^ Since the rise of ISIS and opposition militias in 2014, HCH and ZCC have treated hundreds of internally displaced persons who reside in Sulaymaniyah as well as patients who travel back and forth from their places of origin. Thirty-five percent of HCH's patients are original residents of a province outside the KRG. Oncologists in other KRG cancer centers likewise report large patient populations of displaced persons. The sole blemish on the recent ASCO conference in Erbil came during the opening ceremonies, when several officials drew attention to these developments and publicly lamented shortages arising from the consumption of medical resources by displaced persons.

Such rhetoric does not reflect the attitudes of the vast majority of oncologists who serve within the KRG and treat displaced patients on a daily basis. Moreover, from the standpoint of advancement of cancer research in the KRG, a focus solely on the so-called burden of the displaced population misses an important opportunity to improve the oncology capacities and care delivery models in the region. Displaced persons who undergo oncology treatments in the KRG possess vast knowledge and experience about the strategies of access to cancer care under complex conditions of war. Their narratives can provide essential data for teams of oncologists and researchers to develop better models of care, which would benefit not only the KRG but also other major hubs for displaced patients (eg, Baghdad, Erbil, and Kirkuk).

Ghassan Abu Sitta, Omar Dewachi, and a team of other physicians who specialize in the emerging field of conflict medicine have written: “In war-torn environments, where data collection is nearly impossible, patients’ narratives … will help in better understanding the ways affected families and communities strategize their survival by triaging their health needs with the resources and facilities available to them.”^10^ The broader point by Abu-Sitta et al is that an interdisciplinary approach to research is essential in contexts of war. Social scientists, epidemiologists, and others must team up with oncologists to understand the complex dynamics of cancer care delivery. By adopting an interdisciplinary approach to research, oncology hospitals in the KRG and Iraq could view themselves as centers for developing models of health care for populations undergoing oncology treatments under long-term conditions of war and conflict.

## Initial Study

As an initial step for implementing this agenda, we recently began a research project to understand the experiences of patients undergoing treatments in the KRG who are either internally displaced or unable to acquire cancer treatments in their places of origin. The first phase of the project is based at the HCH in Sulaymaniyah, where 75 such patients and/or caregivers are being interviewed. The majority of these patients are Sunni Arabs who fled to the KRG during 2014. They hail from Anbar, Salah-a-din, Mosul, and Diyala. The study goal is to understand how these patients and their families have navigated access to cancer care under conditions of war, insecurity, and displacement. Subsequent interviews will also be conducted in the main Kirkuk and Erbil cancer centers.

In addition to collection of socioeconomic data and information about treatment expenditures, the study involves survey methodology that includes two matrices. One matrix tracks the care-seeking itinerary; that is, it documents the location, time period, and modality of treatment from the onset of the disease to the present. The second matrix tracks the pathways of internal displacement, that is, all of the temporary or semipermanent sites of residence since the time patients left their original homes. The interviews also include a qualitative section to ensure that patients and caregivers have the opportunity to interpret and explain the matrix data in their own words. Analysis of the data from the two matrices together will enable an understanding of how displacement and care-seeking pathways are intertwined.

Although data collection is still underway, preliminary results have taken us aback. Participant responses and personal narratives often overwhelm with the flood of details: visits to dozens upon dozens of hospitals across many different cities because of shifts in security conditions and availability of care; houses, jewelry, and properties sold to finance care in a mix of public and private clinics; shuttled pharmaceuticals across provincial and international borders; impeded access to hospitals because of overly restrictive security measures at checkpoints that cast suspicion on the internally displaced persons who flee ISIS-controlled areas. Such responses speak to the unique challenges of administering care in a region of war and displacement and, ultimately, to the need for collaboration among hospitals, security officials, and other stakeholders.

Although recent studies have explored the nexus between cancer care and conflict,^11^ it remains an area that is little understood in the fields of cancer-related research. Given that the population of displaced patients in the KRG is growing each month,^9^ an understanding of these conflict dynamics will be important to ensure that the KRG can improve models of care.

What are the long-term implications of this argument? Analysts and government officials have begun to speak of what is to come in the post-ISIS period and of the return of the displaced to their homes,^12^ but the reality is that the KRG cancer care system will administer care to a large displaced population for years, if not decades, to come. Cancer requires a complex array of treatment modalities, and many displaced families have told us that they cannot justify a return to provinces where the already fragile health infrastructure will require years of repair, even if the conditions are livable from safety and security standpoints. KRG oncologists likely will need to conceptualize what it will mean to serve this population for a long period. Interdisciplinary research studies like the one described in this commentary will ensure continual improvement toward this goal.

In conclusion, we caution that notions of progress in cancer care imported from other contexts may not necessarily provide useful frameworks for the KRG and Iraq. In the United States, for example, the overarching research priority of the global war on cancer since the 1970s has been to find a cure and decisively defeat the disease.^13^ This absolute and resolute focus on combat against the disease itself does not fit the reality of oncology in the KRG and Iraq, where textbook-perfect protocols are upended repeatedly by the shifting realities of conflict and displacement that face patients. As the KRG looks into the future and continues to improve treatment models, more holistic terminologies may be needed that focus less narrowly on battling the disease and more broadly on ensuring access to care,^14^ including for those most affected by ongoing conditions of conflict.
